# Filippi syndrome-associated CKAP2L modulates microtubule dynamics essential for mitosis and ciliary length regulation

**DOI:** 10.1093/jmcb/mjaf054

**Published:** 2025-12-10

**Authors:** Qian Lyu, Yinghao Wang, Jun Zhou, Huijie Zhao, Tao Zhong, Qingchao Li

**Affiliations:** Center for Cell Structure and Function, College of Life Sciences, Shandong Normal University, Jinan 250014, China; Center for Cell Structure and Function, College of Life Sciences, Shandong Normal University, Jinan 250014, China; Center for Cell Structure and Function, College of Life Sciences, Shandong Normal University, Jinan 250014, China; State Key Laboratory of Medicinal Chemical Biology, Haihe Laboratory of Cell Ecosystem, College of Life Sciences, Nankai University, Tianjin 300071, China; Center for Cell Structure and Function, College of Life Sciences, Shandong Normal University, Jinan 250014, China; Institute of Immunology and Molecular Medicine, Key Laboratory of Cell and Biomedical Technology of Shandong Province, College of Basic Medicine, Jining Medical University, Jining 272067, China; Center for Cell Structure and Function, College of Life Sciences, Shandong Normal University, Jinan 250014, China

**Keywords:** CKAP2L, microtubule, mitosis, centrosome, cilia

## Abstract

Mutations in the gene encoding cytoskeleton-associated protein 2-like (CKAP2L) have been identified as a causative factor for Filippi syndrome, a rare developmental disorder characterized by facial dysmorphism, syndactyly, and microcephaly. However, the cellular and molecular mechanisms by which CKAP2L contributes to the pathogenesis of this syndrome remain largely unknown. Here, we generated a *Ckap2l* knockout mouse model to investigate the *in vivo* and cellular roles of CKAP2L. Interestingly, *Ckap2l* knockout mice show no overt developmental abnormalities, with the exception of reduced male fertility, evidenced by decreased sperm count, impaired motility, and abnormally elongated flagella. At the cellular level, CKAP2L is a *bona fide* microtubule-associated protein that localizes to microtubule-based organelles, including the centrosome, mitotic spindle, and ciliary basal body. Depletion of CKAP2L leads to shortened mitotic spindles and cytokinesis failure, resulting in multinucleation. Furthermore, we uncover a conserved function for CKAP2L as a negative regulator of primary cilium length; its loss markedly increases ciliary length in both human and mouse cells. Collectively, these findings position CKAP2L as a multifunctional regulator of microtubule-based organelles and suggest that Filippi syndrome can be classified as a ‘centrosomopathy’ arising from concurrent defects in cell proliferation and ciliary function.

## Introduction

A precisely coordinated interplay between microtubule dynamics, mitotic spindle assembly, and cell cycle checkpoints is indispensable for normal development and tissue homeostasis (Janke and Magiera, 2020; Sun et al., 2025). Disruption of this orchestration not only compromises organogenesis but also fuels chromosomal instability, a molecular hallmark of many cancers (Sferra et al., 2020). Cytoskeleton-associated protein 2-like (CKAP2L), a microtubule-binding protein encoded on chromosome 2, has recently emerged as a pivotal component of this regulatory network (Yumoto et al., 2013; Hussain et al., 2014). CKAP2L is a 745-amino acid microtubule-binding protein, characterized principally by a C-terminal CKAP2 domain (amino acids 415–734) and an anaphase-promoting complex/cyclosome (APC/C)-binding KEN box degron motif (Lys–Glu–Asn, amino acids 185–187) (Yumoto et al., 2013). Loss of CKAP2L in cultured cells results in aberrant spindle assembly, consequently impairing chromosome segregation (Hussain et al., 2014). Conversely, CKAP2L overexpression can lead to spindle abnormalities and induce cell cycle arrest (Yumoto et al., 2013). In addition, CKAP2L directly interacts with RNA polymerase II and has been implicated in regulating processes such as spindle assembly checkpoint formation, chromosome segregation, and E2F signal transduction (Monteverde et al., 2021). Although existing studies indicate that CKAP2L deficiency severely disrupts mitosis, the precise molecular mechanisms governing CKAP2L’s role in mitotic regulation remain largely elusive.

Beyond mitosis, microtubules form the structural core of cilia—the hair-like organelles. Functionally, cilia are classified into primary and motile cilia. In higher animals, motile cilia are often found in clusters on the surface of epithelial cells, such as those lining the trachea, the ependyma of brain ventricles, and the fallopian tubes. Through their coordinated beating, motile cilia generate fluid flow across the cell surface, serving functions like maintaining airway clearance and hydration, propelling cerebrospinal fluid, and facilitating the transport of oocytes/zygotes (Yan et al., 2016; Lyu et al., 2024; Song et al., 2025; Zhu, 2025). Primary cilia are typically solitary and non-motile and primarily function as sensory organelles. They perceive extracellular stimuli via receptors and ion channels embedded in the ciliary membrane, transducing these signals into intracellular cascades (May-Simera and Kelley, 2012; Basten and Giles, 2013; Zimmerman and Yoder, 2015; Bangs and Anderson, 2017; Tian et al., 2023; Zhao et al., 2023). For instance, during embryonic development, primary cilia play crucial roles in mediating signaling pathways essential for development, including Sonic Hedgehog (Shh) and Wnt pathways (May-Simera and Kelley, 2012; Abraham et al., 2022; Schmidt et al., 2022; Szymanska et al., 2022). Consequently, defects in ciliary structure or function lead to a spectrum of human genetic disorders collectively known as ciliopathies (Reiter and Leroux, 2017; Focsa et al., 2021; Li et al., 2024; Lyu et al., 2024). The clinical manifestations of ciliopathies include short stature, craniofacial abnormalities, skeletal dysplasia, polydactyly, intellectual disability, obesity, infertility, and polycystic kidney disease (Reiter and Leroux, 2017; Anvarian et al., 2019; Wallmeier et al., 2020; Andreu-Cervera et al., 2021).

Filippi syndrome is an exceedingly rare autosomal recessive genetic disorder, with <40 cases documented worldwide (Capecchi et al., 2018). The cardinal features of Filippi syndrome include microcephaly, pre- and postnatal growth restriction, syndactyly, and distinctive craniofacial features (Hussain et al., 2014). At the molecular level, mutations in the *CKAP2L* gene are the only currently established genetic cause of Filippi syndrome (Hussain et al., 2014; Capecchi et al., 2018; Patrick et al., 2022). Given that impairments in ciliary structure or function are known to impact skeletal and nervous system development (Youn and Han, 2018; Park et al., 2019; Lim et al., 2020; Chinipardaz et al., 2022; Li et al., 2022), we hypothesize that CKAP2L, as a microtubule-binding protein, may contribute to the pathogenesis of Filippi syndrome by influencing both mitosis and ciliary function, thereby playing a role in regulating skeletal and nervous system development. Here, we dissect the function of CKAP2L from organism to organelle. We demonstrate that its loss in mice reduces male fertility due to flagellar defects, and, at the cellular level, reveal its essential roles in microtubule nucleation, mitotic spindle assembly, and ciliary length control. Our findings provide a unified mechanistic framework for the diverse pathologies of Filippi syndrome.

## Results

### Ckap2l deficiency impairs male fertility in mice

**Figure 1:**
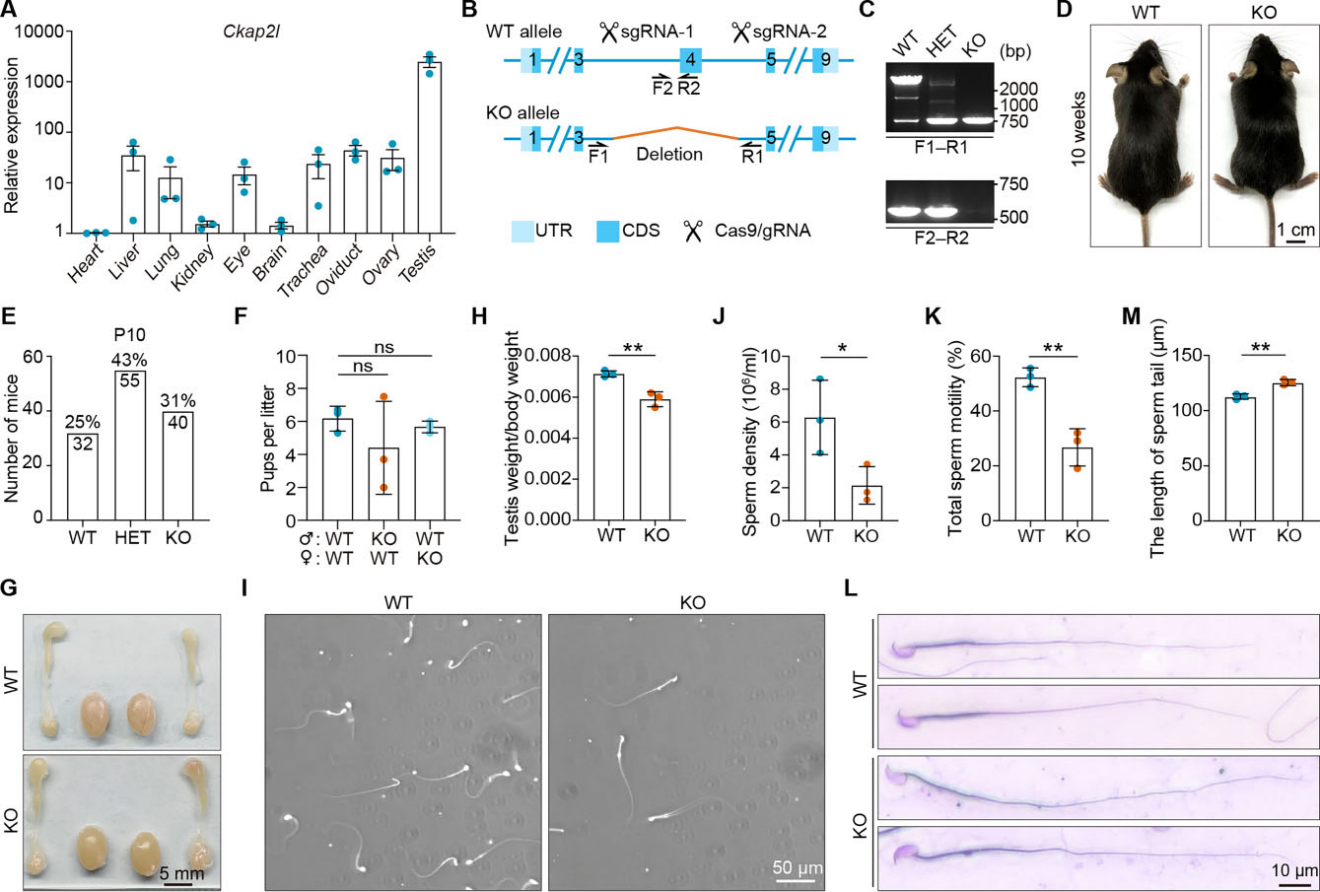
Loss of *Ckap2l* in mice causes a reduction in male fertility and defects in sperm flagella. (**A**) qRT-PCR analysis of *Ckap2l* mRNA expression across various mouse tissues, normalized to *Gapdh* and baseline 1 (heart) (△△C_T_ method). Data are presented as mean ± SEM (*n =* 3). (**B**) Schematic of the CRISPR–Cas9 strategy used to generate the *Ckap2l* KO allele by deleting exon 4. Locations of sgRNAs and genotyping primers (F1, R1, F2, R2) are indicated. UTR, untranslated region; CDS, coding sequence. (**C**) Representative PCR genotyping of WT, heterozygous (HET), and KO mice. (**D**) *Ckap2l* KO mice were born at expected Mendelian ratios. (**E**) Gross morphology of an 8-week-old *Ckap2l* KO mouse appears normal compared to a WT littermate. (**F**) Fertility test showing the average number of pups per litter sired by WT and KO males over a 3-month period (*n =* 3 mice per genotype). (**G**) Representative images of testes and epididymides from WT and KO mice. (**H**) Gonadosomatic index (testis weight/body weight) is significantly reduced in KO mice. (**I**) Representative images from CASA. (**J**) Sperm count from the cauda epididymis is drastically reduced in KO mice. (**K**) The percentage of total motile sperm is significantly lower in KO mice. (**L**) Giemsa staining of spermatozoa. (**M**) The flagellar length of KO spermatozoa is significantly increased compared to WT. Data are presented as mean ± SD. Unpaired two-tailed *t*-test was performed. **P < 0.01, *P < 0.05; ns, not significant.

To create a comprehensive overview of *Ckap2l* expression, we examined its tissue distribution pattern in mice. A panel of tissues was collected from 8-week-old wild-type (WT) mice and subjected to quantitative real-time polymerase chain reaction (qRT-PCR) and immunoblotting. As shown in Figure 1A, *Ckap2l* mRNA expression was highest in the testis. This expression pattern suggests that *Ckap2l* may play a role in the male reproductive system.

To define the function of CKAP2L *in vivo*, a *Ckap2l* knockout (KO) mouse model was generated using CRISPR–Cas9 technology with dual single guide RNAs (sgRNAs) (Figure 1B). Genome deletions introduced by sgRNAs were identified by PCR (Figure 1C). *Ckap2l* KO mice were born at Mendelian ratios without gross morphological or behavioral abnormalities (Figure 1D and E).

Given the highest expression of CKAP2L in the testis, we sought to examine the fertility of *Ckap2l* KO males. *Ckap2l* KO and WT males or females were separately mated to C57BL/6J mice, and litter sizes were recorded. Intriguingly, *Ckap2l* KO males (4.4 ± 2.8; *n =* 3) sired fewer offspring than WT males (6.2 ± 0.8; *n =* 3) over a 3-month mating period, although this difference did not reach statistical significance (Figure 1F; *P* = 0.3532), suggesting potential subfertility. Consistently, the gonadosomatic index was significantly reduced (Figure 1G and H; *P <* 0.01). These data indicate that *Ckap2l* deficiency impairs male fertility, demonstrating its essential role in the male reproductive system.

To understand the mechanism underlying declined fertility in *Ckap2l* KO males, computer-assisted sperm analysis (CASA) was conducted to evaluate the motility of sperm extracted from the cauda epididymis of adult WT and *Ckap2l* KO mice. Representative images and videos from CASA illustrate the difference in motility and morphology between WT and *Ckap2l* KO spermatozoa (Figure 1I; Supplementary Video S1). *Ckap2l* KO spermatozoa showed reduced density and disorganized movement paths compared to WT sperm (Figure 1I). The counts of spermatozoa obtained from *Ckap2l* KO cauda epididymis were drastically reduced [(6.3 ± 2.3) × 10^6^/ml (WT) vs. (2.1 ± 1.1) × 10^6^/ml (KO); *P <* 0.05] (Figure 1J). The percentage of total motile *Ckap2l* KO spermatozoa (26.7 ± 6.8; *n =* 3) was also significantly reduced compared with WT spermatozoa (52.3 ± 3.4; *n =* 3) (Figure 1K; *P <* 0.01). Interestingly, both CASA and Giemsa analyses revealed a significant increase in flagellar length in *Ckap2l* KO spermatozoa [112.7 ± 2.4 μm (WT) vs. 122.1 ± 8.5 μm (KO); *P <* 0.01] (Figure 1I, L, and M), indicative of teratospermia in *Ckap2l* KO males. These data demonstrate that CKAP2L is essential for male fertility in mice.

### CKAP2L localizes to centrosomes, basal bodies, and cilia

**Figure 2:**
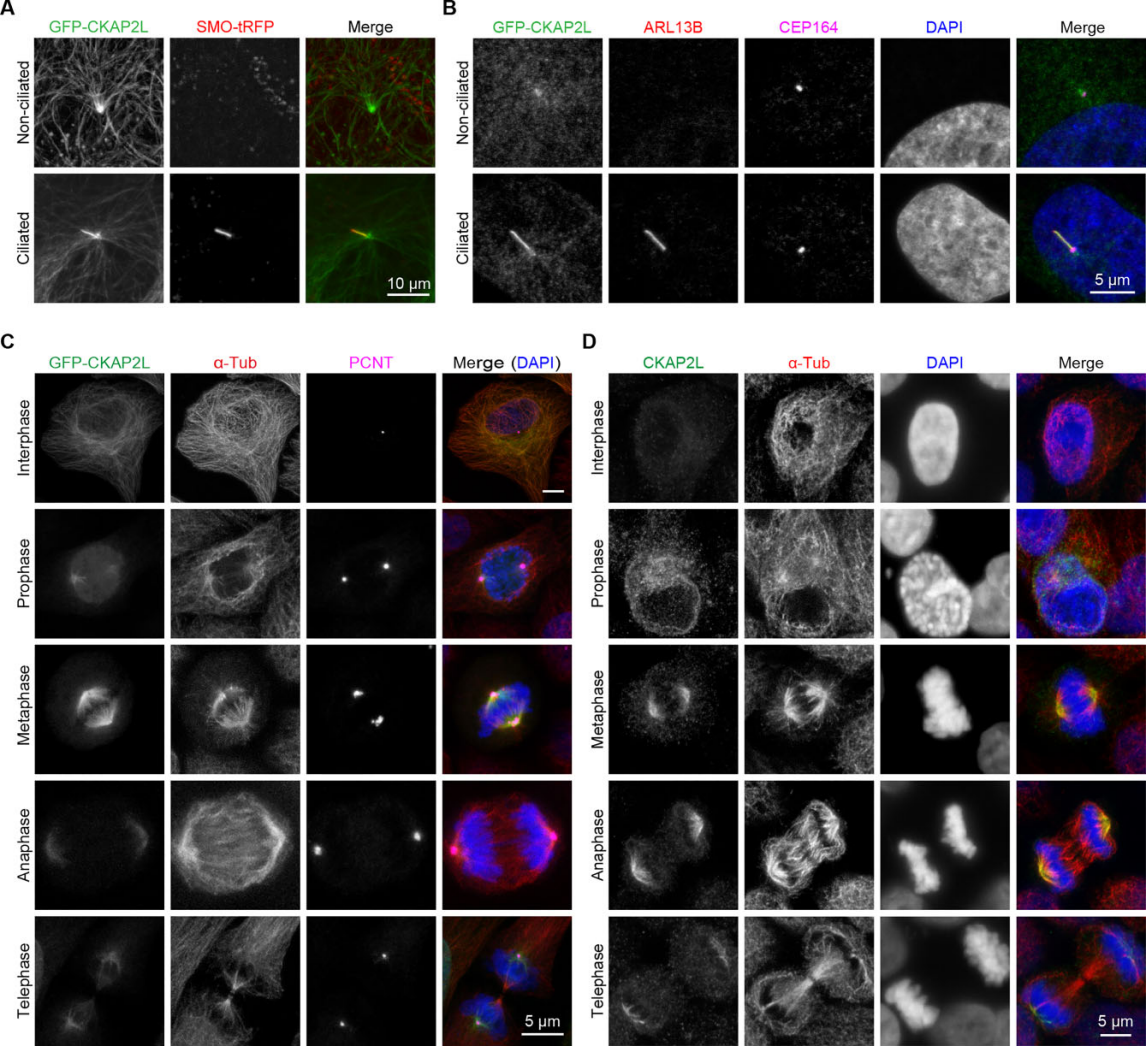
CKAP2L is a MAP that localizes to the centrosome, cilium, and mitotic spindle. (**A**) Live-cell imaging of RPE1 cells co-expressing GFP-CKAP2L (green) and SMO**-**tRFP (red). Note that GFP-CKAP2L localizes to cytoplasmic microtubules and is enriched at the centrosome, basal body, and ciliary axoneme. (**B**) Immunofluorescence of RPE1 cells expressing GFP-CKAP2L (green), co-stained for the cilium (ARL13B, red), the basal body/mother centriole (CEP164, magenta), and DNA (DAPI, blue). (**C** and **D**) Immunofluorescence images of HeLa cells expressing GFP-CKAP2L (**C**) and HeLa cells (**D**) at different stages of the cell cycle. Microtubules are labeled with α-tubulin (α-Tub, red), and DNA is stained with DAPI (blue).

To investigate CKAP2L’s cellular functions, we analyzed its subcellular localization. GFP-CKAP2L was co-expressed with a ciliary marker SMO-tRFP in human RPE1 cells and live imaged under normal (non-ciliated) and serum-starved (ciliated) conditions (Lu et al., 2015). Live-cell imaging showed that GFP-CKAP2L localizes to microtubules in both non-ciliated and ciliated cells and accumulates at the basal body and ciliary axoneme in ciliated cells (Figure 2A). To further confirm its localization, we used ARL13B and CEP164 antibodies to label cilia and the mother centriole, respectively. Immunostaining of RPE1 cells expressing GFP-CKAP2L confirmed its localization at the centrosome in non-ciliated cells, as well as at the basal body and ciliary axoneme in ciliated cells (Figure 2B).

Since cytoplasmic microtubules form the mitotic spindle during cell division, we examined CKAP2L’s localization during mitosis by performing immunofluorescence analysis in HeLa cells. Consistently, both exogenously expressed GFP-CKAP2L and endogenous CKAP2L were observed at spindle poles and fibers throughout mitosis, with pronounced spindle pole enrichment from prophase to telophase (Figure 2C and D). Notably, overexpressed GFP-CKAP2L displayed stronger microtubule distribution during interphase (Figure 2C), likely due to the higher protein levels. Overall, these findings demonstrate that CKAP2L localizes to cytoplasmic microtubules, centrosomes, and cilia during interphase and accumulates at spindle poles and fibers during mitosis, indicating its role in microtubule-associated organelles.

### CKAP2L binds to and promotes microtubule polymerization

**Figure 3:**
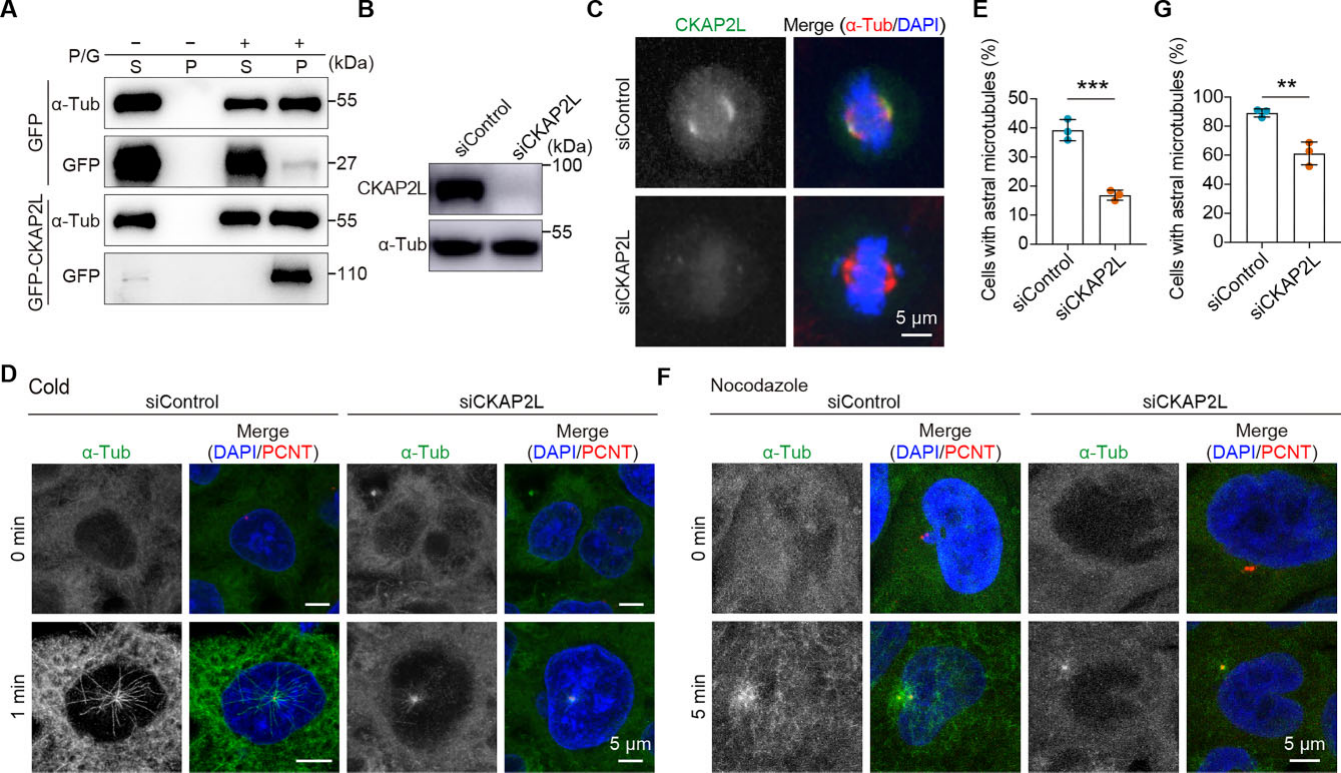
Loss of CKAP2L impairs microtubule nucleation. (**A**) Microtubule co-sedimentation assay. Lysates from cells expressing GFP or GFP-CKAP2L were incubated with (+) or without (–) Taxol and GTP (P/G). After centrifugation, supernatant (S) and pellet (P) fractions were immunoblotted for GFP and α-tubulin. Note that GFP-CKAP2L but not GFP co-sediments with microtubules. (**B**) Immunoblotting of HeLa cells showing efficient siRNA-mediated knockdown of endogenous CKAP2L. GAPDH serves as a loading control. (**C**) Immunofluorescence analysis showing the loss of CKAP2L in siCKAP2L-treated mitotic cells. Cells were stained with CKAP2L (green) and α-Tub (red) antibodies and DAPI (blue). (**D**–**G**) Microtubule regrowth assay. Microtubules were depolymerized with cold treatment (**D** and **E**) or nocodazole (**F** and **G**). The formation of microtubule asters was induced by either 1-min rewarming or 5-min nocodazole washout. Cells were stained with PCNT (red) and α-Tub (green) antibodies and DAPI (blue). More than 80 cells from three independent experiments were analyzed. Data are presented as mean ± SD. Unpaired two-tailed *t*-test was performed. ***P < 0.001, **P < 0.01.

Given the localization of CKAP2L to microtubule-associated structures, we performed the *in vitro* microtubule co-sedimentation assay to determine whether CKAP2L is a *bona fide* microtubule-associated protein (MAP). Lysates from HEK293T cells expressing either GFP-CKAP2L or GFP were incubated with Taxol to stabilize microtubules. Following centrifugation, the supernatant (S) and pellet (P) fractions were analyzed by immunoblotting. As shown in Figure 3A, the majority of α-tubulin sedimented into the pellet, confirming successful microtubule polymerization. While GFP was exclusively in the supernatant, GFP-CKAP2L was found primarily in the pellet fraction (Figure 3A), indicating that CKAP2L binds to polymerized microtubules.

To investigate the cellular role of CKAP2L, we knocked down its expression in HeLa cells using a specific small interfering RNA (siRNA) (Wang and He, 2020). Immunoblotting and immunostaining confirmed efficient depletion of CKAP2L in siCKAP2L-treated cells compared to the control sample (siControl) (Figure 3B and C). To determine the role of CKAP2L in microtubule nucleation or stability, we performed microtubule regrowth assays. Microtubules were depolymerized with cold treatment. Upon rewarming after cold shock, cells treated with siControl rapidly nucleated dense aster microtubules from the pericentrin (PCNT)-marked centrosomes within 1 min (Figure 3D). In contrast, CKAP2L depletion severely impaired microtubule regrowth, with only a few short microtubules forming at the centrosome in siCKAP2L-treated cells (Figure 3D and E). These results were confirmed with a nocodazole-washout assay. Microtubules were depolymerized by nocodazole exposure, and 5 min after nocodazole removal, control cells regrew prominent asters, whereas CKAP2L-depleted cells again failed to do so effectively (Figure 3F and G).

To explore how CKAP2L promotes microtubule assembly, we examined whether CKAP2L interacts with core components of the microtubule nucleation machinery, γ-tubulin and NEDD1 (Sulimenko et al., 2022; Ali et al., 2023; Munoz-Hernandez et al., 2025). By performing immunoprecipitation of GFP-CKAP2L from HEK293T cell lysates, we did not detect strong interactions between CKAP2L and either γ-tubulin or NEDD1 (Supplementary Figure S1), suggesting that CKAP2L likely functions independently of γ-tubulin and NEDD1. Collectively, these data demonstrate that CKAP2L is a centrosome-localized MAP required for efficient microtubule nucleation.

### CKAP2L regulates mitotic spindle assembly and proper cytokinesis

**Figure 4:**
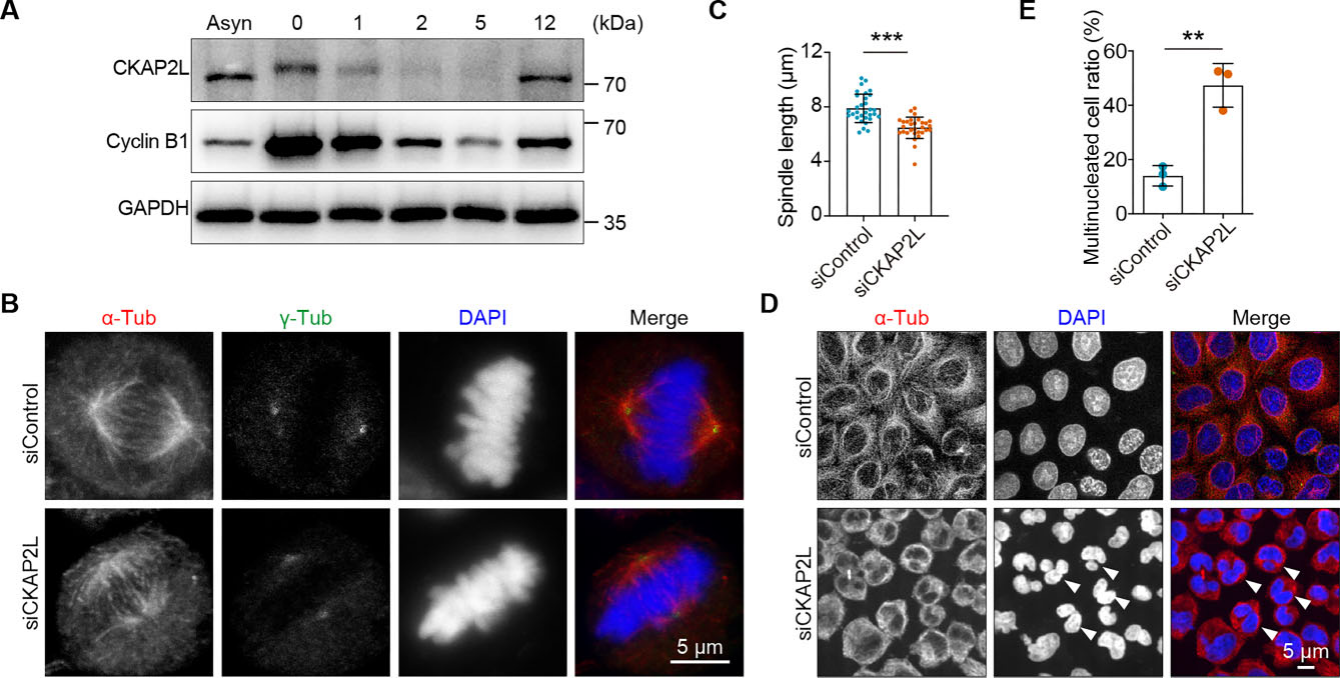
CKAP2L deficiency affects proper spindle formation and causes cytokinesis failure. (**A**) Immunoblotting of synchronized HeLa cells at different cell cycle stages. CKAP2L protein levels vary throughout the cell cycle, peaking in mitosis and decreasing after mitotic exit, mirroring the expression pattern of Cyclin B1. GAPDH serves as the loading control. (**B**) Representative Immunofluorescence images of mitotic spindles in siControl- and siCKAP2L-treated cells. Note that the spindles in CKAP2L-depleted cells appear shorter. Cells were stained with α-Tub (red) and γ-Tub (green) antibodies and DAPI (blue). (**C**) Quantification of the pole-to-pole length of spindles. (**D**) Immunofluorescence images of siControl- and siCKAP2L-treated HeLa cells stained for α-Tub (red) and DNA (blue). Arrowheads indicate multinucleated cells. (**E**) Quantification of the percentage of multinucleated cells. Data are presented as mean ± SD (*n >* 80 cells from three independent experiments). Unpaired two-tailed *t*-test was performed. ***P < 0.001, **P < 0.01.

Given the critical role of CKAP2L in microtubule nucleation, we sought to investigate its involvement in cell division. Immunoblotting of cells synchronized at different cell cycle stages revealed that CKAP2L levels were not constant but fluctuated significantly, increasing as cells progressed toward division (Figure 4A). This expression pattern closely resembled that of Cyclin B1, a well-known mitotic marker (Strauss et al., 2018; Zheng et al., 2024), indicating a functional role for CKAP2L during cell division. To test this, we examined the mitotic spindle structure in control and CKAP2L-depleted cells. Mitotic spindles and spindle poles were labeled with α-tubulin and γ-tubulin, respectively. Interestingly, although bipolar spindles with chromosomes properly aligned at the metaphase plate could be observed in both control and CKAP2L-depleted cells, the spindles in siCKAP2L-treated cells appeared much shorter and more compact (Figure 4B). Quantification of the pole-to-pole distance of the spindles revealed a significant reduction in the spindle length in CKAP2L-depleted cells [7.9 ± 1.0 μm (siControl) vs. 6.5 ± 0.8 μm (siCKAP2L); *P <* 0.001] (Figure 4C). These data suggest that CKAP2L is required for establishing or maintaining the proper length of the mitotic spindle.

Defects in the mitotic spindle structure frequently cause cytokinesis failures. Therefore, we examined the overall cellular morphology in asynchronous populations of control and CKAP2L-depleted cells. Notably, immunostaining showed a significant increase in the number of multinucleated cells in siCKAP2L-treated cells (Figure 4D, white arrowheads). Quantification revealed that >45% of siCKAP2L-treated cells were multinucleated, compared to ~14% of the control cells (Figure 4E; *P <* 0.01). These findings highlight the essential role of CKAP2L in maintaining mitotic spindle integrity and ensuring proper cell division.

### CKAP2L depletion causes excessive elongation of primary cilia

**Figure 5:**
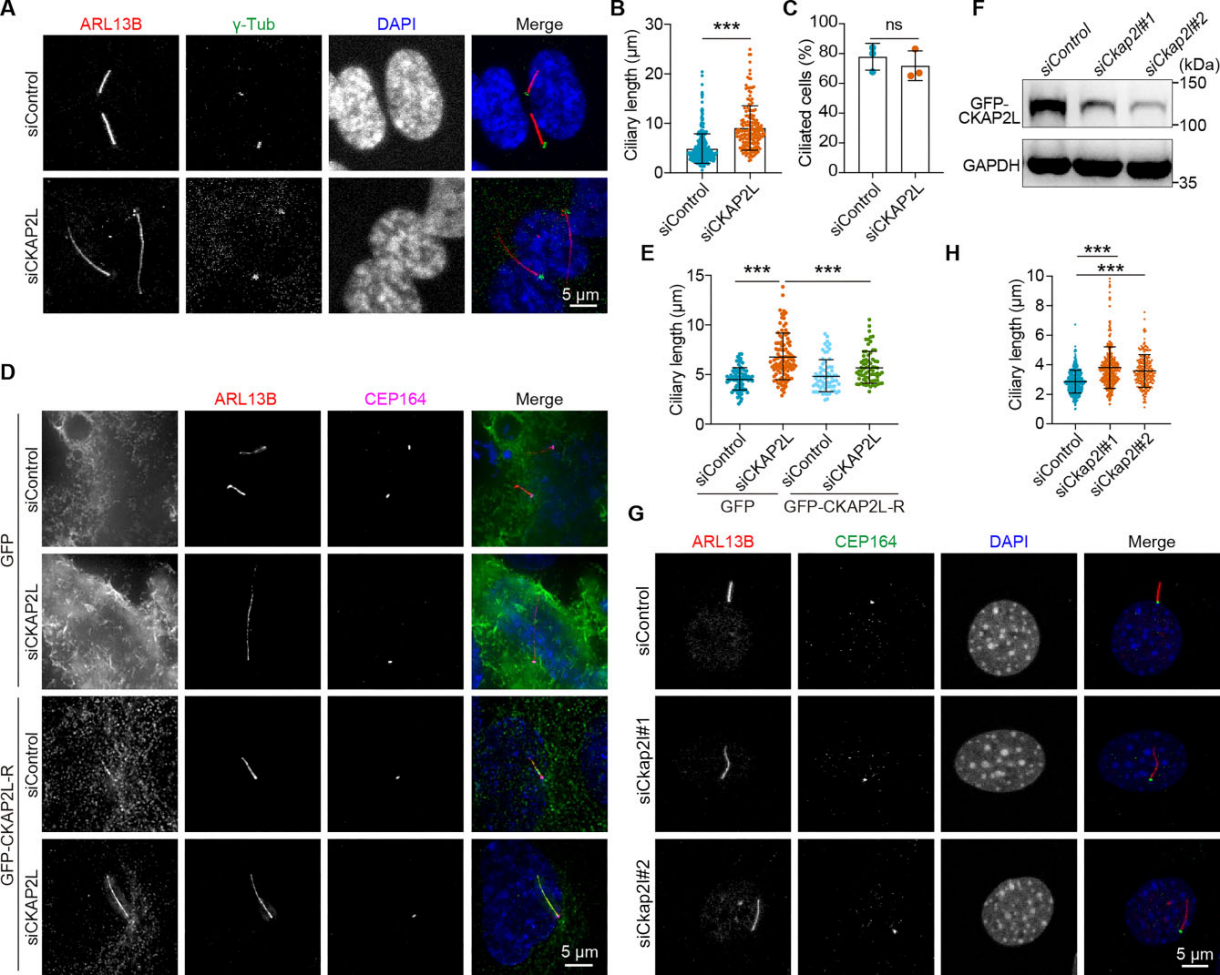
CKAP2L is a conserved negative regulator of ciliary length. (**A**) Immunofluorescence images of primary cilia in serum-starved RPE1 cells treated with siControl or siCKAP2L oligo. Cilia and basal bodies are marked by ARL13B (red) and γ-Tub (green), respectively. (**B** and **C**) Quantification of ciliary length (**B**) and the percentage of ciliated cells (**C**). (**D** and **E**) Rescue experiment in RPE1 cells. Note that ciliary elongation caused by siCKAP2L can be rescued by co-expression of an siRNA-resistant GFP-CKAP2L-R, but not by GFP. (**D**) Cilia are marked by ARL13B (red), basal bodies by CEP164 (magenta), and transfected cells by GFP (green). (**E**) Quantification of the ciliary length. (**F**) Immunoblotting of mouse NIH-3T3 cells confirming that the two independent siRNA oligos (siCkap2l#1 and siCkap2l#2) could efficiently recognize and inhibit the expression of GFP-fused mouse Ckap2l. Each siRNA oligo was co-transfected with a construct expressing GFP-fused mouse Ckap2l. Cells were then lysed and subjected to immunoblotting. GAPDH serves as a loading control. (**G**) Immunofluorescence images of primary cilia in serum-starved NIH-3T3 cells treated with siControl or either siCkap2l oligo. Cilia and basal bodies are marked by ARL13B (red) and CEP164 (green), respectively. (**H**) Quantification of the ciliary length in NIH-3T3 cells. Data are presented as mean ± SD (*n >* 200 cilia from three independent experiments). Unpaired two-tailed *t*-test was performed. ***P < 0.001; ns, not significant.

Given the essential role of microtubules in centrosomes and primary cilia, we further examined the effects of CKAP2L depletion on primary cilia in RPE1 cells. Immunofluorescence analysis using the ciliary marker ARL13B revealed that while both control and siCKAP2L-treated cells formed primary cilia, the cilia in the CKAP2L-depleted cells were significantly longer than those in control cells [4.9 ± 3.0 μm (siControl) vs. 9.1 ± 4.5 μm (siCKAP2L); *P <* 0.001] (Figure 5A–C). These results suggest that CKAP2L is not necessary for the initial formation of the cilium (ciliogenesis) but, instead, has a role in regulating ciliary length.

To confirm that the observed ciliary over-elongation was specifically caused by CKAP2L loss rather than an off-target effect of the siRNA, we conducted a rescue experiment. We co-transfected cells with CKAP2L siRNA oligos and a construct expressing the siRNA-resistant, GFP-tagged CKAP2L (GFP-CKAP2L-R). As shown in Figure 5D and E, expression of GFP in CKAP2L-depleted cells had no apparent effect on elongated cilia. However, expression of GFP-CKAP2L-R could partially rescue the phenotype, restoring ciliary length to that observed in control cells. This result confirms that the ciliary length defect is specifically attributable to the loss of CKAP2L.

To determine whether this function of CKAP2L is conserved across species, we performed knockdown experiments in mouse NIH-3T3 cells using two different siRNA oligos (siCkap2l#1 and siCkap2l#2). Western blot analysis confirmed that both siRNA oligos could efficiently recognize and inhibit the expression of GFP-fused mouse Ckap2l (Figure 5F). Consistently, the loss of CKAP2L in NIH-3T3 cells also resulted in a significant elongation of primary cilia (Figure 5G and H). To determine whether CKAP2L influences ciliary length by regulating intraflagellar transport (IFT), we assessed the cellular distribution of core IFT proteins. Immunofluorescence analysis showed that the distribution of IFT-B (IFT88 and IFT81) and IFT-A (IFT140) proteins along the ciliary axoneme was not overtly affected in CKAP2L-depleted cells, despite significant ciliary elongation (Supplementary Figure S2). These findings suggest that CKAP2L is a conserved, negative regulator of primary cilium length.

## Discussion

The centrosome’s ability to organize the interphase cytoskeleton, form the mitotic spindle, and template the primary cilium is fundamental to cellular life and organismal development. Our findings identify CKAP2L, a protein previously implicated in the rare genetic disorder Filippi syndrome (Capecchi et al., 2018), as a MAP that can operate at the nexus of these three fundamental cellular processes.

The centrosome serves as the primary microtubule-organizing center (MTOC) in most animal cells (Jaiswal et al., 2021). The direct binding of CKAP2L to microtubules and its specific localization to the centrosome provide a strong foundation for its function. Microtubule regrowth assays showed a severe impairment in the ability of centrosomes to nucleate microtubules upon CKAP2L depletion. Given that no stable interaction was detected between CKAP2L and the core γ-tubulin ring complex (γ-TuRC) components, γ-tubulin and NEDD1, CKAP2L probably is not a direct or stable component of the nucleation complex. Instead, it more likely functions to protect or stabilize microtubules as they emerge from the centrosome, thereby promoting the formation of a robust aster. The precise mechanism by which CKAP2L promotes microtubule nucleation remains to be elucidated.

A properly scaled and functional spindle is a prerequisite for the accurate signaling that defines the cleavage plane and executes cell scission during cell division. Our study confirms and extends previous findings that CKAP2L is critical for mitosis. The cell cycle-dependent expression of CKAP2L, which peaks in mitosis in concert with Cyclin B1, is indicative of its specialized role during cell division. We show that loss of CKAP2L leads to significantly shorter mitotic spindles. This structural defect likely causes subsequent cytokinesis failure, as evidenced by a high percentage of multinucleated cells. The observed phenotype suggests that CKAP2L may regulate the stability or dynamics of interpolar microtubules that determine spindle length, a function consistent with its identity as a MAP. These findings establish a mechanistic connection between CKAP2L and maintaining genomic stability.

A key and novel finding of this work is the role of CKAP2L as a negative regulator of primary cilium length. Ciliary length is dynamically maintained by a balance between assembly and disassembly rates (Keeling et al., 2016; Macarelli et al., 2023). In both human and mouse cells, depletion of CKAP2L resulted in significantly elongated cilia without affecting the initial ciliogenesis, suggesting it may either promote ciliary disassembly or act as a brake on assembly. Importantly, this function appears to be conserved in regulating the flagellar length of sperm, which are essentially long, motile cilia. The abnormally elongated sperm flagella in *Ckap2l* KO mice impair flagellar motility, leading to a decrease in male fertility. This provides strong *in vivo* evidence for CKAP2L’s role as a conserved regulator of axonemal length. Since no changes in IFT distribution along the cilia were detected upon CKAP2L loss, CKAP2L may not regulate the overall recruitment or stable localization of the IFT machinery (Broekhuis et al., 2013; Wang et al., 2021; Sun et al., 2024). Instead, it may subtly influence the kinetics of IFT, regulate tubulin turnover at the ciliary tip, or operate through an alternative pathway that acts as a length-limiting checkpoint.

The diverse functions of CKAP2L converge at the centrosome, an organelle that undergoes a remarkable transition from an MTOC to a spindle pole during mitosis or a basal body that templates the cilium (Kobayashi and Dynlacht, 2011). Our findings suggest that Filippi syndrome is, at its core, a ‘centrosomopathy’, where dysfunction of a single protein, CKAP2L, disrupts multiple functions of this central organelle. This provides a unified model to explain the pleiotropic clinical features of the syndrome (OMIM: #272440), such as microcephaly and skeletal defects, which likely arise from a devastating synergy between proliferative defects and aberrant developmental signaling. First, the mitotic defects caused by CKAP2L loss—impaired spindle formation and failed cytokinesis—would compromise progenitor cell proliferation during development, contributing directly to growth retardation and microcephaly. Second, the dysregulation of ciliary length would impair the function of primary cilia as critical signaling hubs (Breunig et al., 2008; Lasser et al., 2018; Youn and Han, 2018; Park et al., 2019; Li et al., 2022). An abnormally long cilium may have altered signaling capacity, disrupting these developmental programs.

A crucial question is why the *Ckap2l* KO mouse model does not fully recapitulate the severe human phenotype. Several factors could contribute to this discrepancy. First, genetic redundancy may exist in mice, where a related protein partially compensates for CKAP2L’s absence during critical developmental stages, which may be less effective in humans. Second, there may be species-specific differences in the reliance on CKAP2L in key progenitor cell populations, such as those in the developing brain or limb buds. The mitotic and ciliary defects that we observed might fall below a critical threshold required to cause gross developmental abnormalities in mice but are sufficient to impair the highly specialized and demanding process of spermatogenesis. Finally, some human mutations may result in truncated, misfolded proteins with dominant-negative effects, which would not be captured by a simple null allele. A deeper understanding of CKAP2L’s expression and function in relevant human versus mouse progenitor cells will be necessary to fully resolve this important point.

In conclusion, our study identifies CKAP2L as a master regulator of the centrosome’s functional plasticity, essential for microtubule nucleation, mitotic spindle integrity, and ciliary length control. This work not only advances our fundamental understanding of how cell architecture is controlled but also provides a comprehensive molecular framework for a complex human genetic disorder, linking the fields of cell division, ciliopathy, and developmental disease. Furthermore, as centrosome amplification and mitotic errors are hallmarks of cancer, understanding how proteins like CKAP2L ensure mitotic fidelity and genomic stability may offer new insights into tumorigenesis.

## Materials and methods

### Plasmids

Full-length human *CKAP2L* (NM_152515) was obtained from the DNASU Plasmid Repository (Arizona State University). The full-length fragment was PCR-amplified and subcloned into the recombinational donor vector pDONR221 to generate entry clones (11789100, ThermoFisher). LR recombination reactions between entry clones and desired gateway destination vectors (Kit #1000000211, Addgene) (Wall et al., 2014) were performed to generate the expression constructs (11791100, ThermoFisher). All constructs were verified via Sanger sequencing analysis. All the primers used are listed in Supplementary Table S1.

### Cell culture and transfection

HEK293T, HeLa, and NIH-3T3 cells were cultured in DMEM (Thermo Fisher, C11995500BT) supplemented with 10% fetal bovine serum (FBS; Biological Industries, 04–001-1ACS) and 1% penicillin/streptomycin (Solarbio, P1400). RPE1 cells were cultured in DMEM/F-12 (Thermo Fisher, C11330500BT) with 10% FBS and 10 μg/ml hygromycin B (Yeasen Biotech, 60224ES03). All cells were maintained at 37°C and 5% CO_2_ and routinely tested for mycoplasma. Plasmids were transfected using PEI (Polysciences) or Lipofectamine 2000 (Thermo Fisher). siRNAs (50 nM) were transfected using Lipofectamine RNAiMAX (Thermo Fisher). All siRNA and primer sequences are listed in Supplementary Table S1.

### Animals

All mouse experiments were performed in accordance with the ethical guidelines of and were approved by the Animal Experimental Ethics Committee of Shandong Normal University (AEECSDNU2022047). The *Ckap2l* KO mouse model (C57BL/6J background) was generated by Shanghai Model Organisms Center, Inc., using the CRISPR–Cas9 system (gRNA1: TAGTGTGGCTGGAAGCACAA; gRNA2: GACGGAGCACTCATCAGGAA) to delete exon 4 of the mouse *Ckap2l* gene (ENSMUSG00000048327). Mice were genotyped by PCR using 2× Taq Plus Master Mix II (Vazyme, P213). For fertility testing, 8-week-old WT and *Ckap2l* KO males (*n =* 3 per genotype) were individually paired with 8-week-old WT C57BL/6J females for three months. Litter size and birth timing were recorded.

### Immunoblotting

Cells were lysed in RIPA buffer supplemented with a protease inhibitor cocktail (Roche). Protein concentrations were determined using a BCA assay (Thermo Fisher Scientific). Equal amounts of protein were separated by SDS–PAGE, transferred to a polyvinylidene difluoride membrane, and blocked for 1 h in 5% non-fat milk in Tris-buffered saline with 0.1% Tween-20 (TBST). Membranes were incubated with primary antibodies overnight at 4°C, followed by incubation with HRP-conjugated secondary antibodies for 1 h at room temperature. Blots were visualized using an enhanced chemiluminescence (ECL) detection system (Millipore).

### Immunofluorescence microscopy

Immunostaining was performed as previously described (Zhao et al., 2013, 2019). In brief, cells grown on glass coverslips were fixed with 4% paraformaldehyde for 15 min, permeabilized with 0.5% Triton X-100 in phosphate-buffered saline (PBS) for 15 min, and blocked with 4% bovine serum albumin in TBST for 1 h. Cells were incubated with primary antibodies overnight at 4°C, followed by incubation with Alexa Fluor-conjugated secondary antibodies (Thermo Fisher Scientific) for 1 h at room temperature. Coverslips were mounted onto glass slides using ProLong Gold Antifade Mountant (Thermo Fisher Scientific). The confocal images were acquired using a Leica TCS SP8 confocal platform equipped with an HCX Plan Apo 63×/1.40 oil immersion objective, with each scanned line averaged four times. Optical sections were captured at 0.5 μm intervals, and z-stack images were processed with maximum intensity projections (Leica Microsystems). All the antibodies used are listed in Supplementary Table S2.

### qRT-PCR

Total RNA was extracted from mouse tissues using the TransZol Up Plus RNA kit (TransGen Biotech). cDNA was synthesized using the one-step RT-gDNA digestion Super Mix (Yeasen Biotech). qRT-PCR was performed using the SYBR Green PCR Kit (Yeasen Biotech). Gene expression was normalized to *Gapdh*, and relative expression was calculated using the ΔΔC_T_ method. Primers are listed in Supplementary Table S1.

### Sperm analysis

Mouse epididymal sperm motility was evaluated as previously described (Zi et al., 2024; Lu et al., 2025). The cauda epididymis was extracted from 8-week-old male mice, and spermatozoa were obtained and suspended in 1 ml of PBS at 37°C for 10 min. A 4-μl aliquot of the suspension was loaded onto a glass counting chamber for analysis of sperm motility utilizing the CASA image system (IVOS II, Hamilton Thorne). For epididymal sperm count, the cauda epididymis was dissected into small fragments using fine-point scissors, and spermatozoa were fully released into 1 ml PBS at 37°C for 30 min. The resulting sperm suspension was appropriately diluted, and the sperm cell concentration was determined using a hemocytometer.

### Microtubule co-sedimentation assay

HEK293T cells transfected with GFP and GFP-CKAP2L plasmids were washed with 3 ml PBS, scraped with PBS, and centrifuged at 1000 rpm for 5 min at 4°C. The supernatant was discarded, and the cell pellet was resuspended in 2 ml of 1× BRB80 (80 mM PIPES, 1 mM MgCl_2_, 1 mM EGTA, pH 6.8) containing protease inhibitors in a 2-ml tube. The suspension was agitated on a shaker for 30 sec, followed by incubation on ice for 5 min, repeated six times. The suspension was transferred to a 2-ml ultracentrifuge tube and centrifuged at 33000× *g* for 1 h at 4°C. Post-centrifugation, each sample was divided into two 1.5-ml EP tubes using a 1-ml syringe, one supplemented with 1 mM GTP and 20 μM Taxol, and the other left untreated. The tubes with GTP and Taxol were incubated in a water bath at 37°C for 1 h, while the untreated tubes were kept on ice for 1 h. Subsequently, the supernatant and pellet obtained after sucrose density gradient centrifugation were subjected to western blot analysis.

### Microtubule regrowth assays

For cold-induced depolymerization, cells on coverslips were incubated on ice for 30 min. Regrowth was initiated by adding warm (37°C) medium, and cells were fixed at the indicated time points. For nocodazole-induced depolymerization, cells were treated with 10 μM nocodazole for 4 h. The drug was washed out with warm medium to initiate regrowth, and cells were fixed at the indicated time points. Fixed cells were processed for immunofluorescence analysis as described above.

### Cell cycle synchronization

HeLa cells were synchronized at the G2/M boundary by treatment with 100 ng/ml nocodazole for 16 h. Mitotic cells were collected by mechanical shake-off and released into fresh, pre-warmed medium. Samples were collected at the indicated time points for immunoblotting.

### Statistical analysis

All experiments were biologically repeated at least three times. For each experiment, 3–5 mice for each genotype were used. One representative picture from 3–5 mice for each genotype was presented for immunostaining. The quantitative results were presented as mean ± standard deviation (SD) or standard error of the mean (SEM). Statistical analyses were conducted using GraphPad Prism. Unpaired two-tailed Student’s *t*-tests were used for comparisons between two groups. Differences were considered significant when *P*-value was <0.05 (*P < 0.05, **P < 0.01, ***P < 0.001). Image analysis and quantification were performed using ImageJ/Fiji software (NIH).

## Supplementary Material

mjaf054_Supplemental_Files

## Notes

**Author contributions:** Q. Li and T.Z. designed the study. Q. Lyu and Y.W. performed experiments. Q. Lyu, Y.W., H.Z., J.Z., T.Z., and Q. Li analyzed the results. Q. Li and Q. Lyu wrote the manuscript. H.Z. and J.Z. supervised and directed the research. All authors discussed the results and commented on the manuscript.
