# Supplementary material for: Filippi syndrome-associated CKAP2L modulates microtubule dynamics essential for mitosis and ciliary length regulation
Source: J Mol Cell Biol. 2025 Dec 10;18:mjaf054. doi: 10.1093/jmcb/mjaf054 (PMC13417012; doi:10.1093/jmcb/mjaf054)
Supplement: mjaf054_Supplemental_Files [file mjaf054_supplemental_files.zip › JMCB-2025-0445.R2_Supplementary material_update.pdf]

Supplementary material

Supplementary Figures

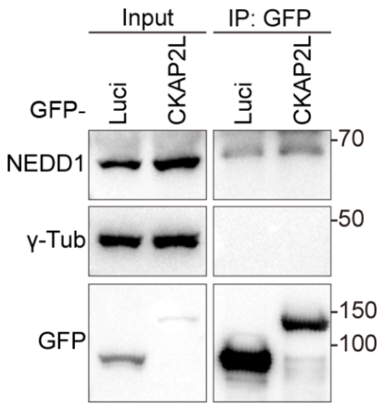

Supplementary Figure S1 CKAP2L does not interact with the γ-TuRC components.

Co-immunoprecipitation of GFP-CKAP2L from HEK293T cell lysates. Lysates from cells expressing GFP-Luci (control) or GFP-CKAP2L were subjected to immunoprecipitation with an anti-GFP antibody. Input and IP fractions were immunoblotted for NEDD1, γ-tubulin, and GFP.

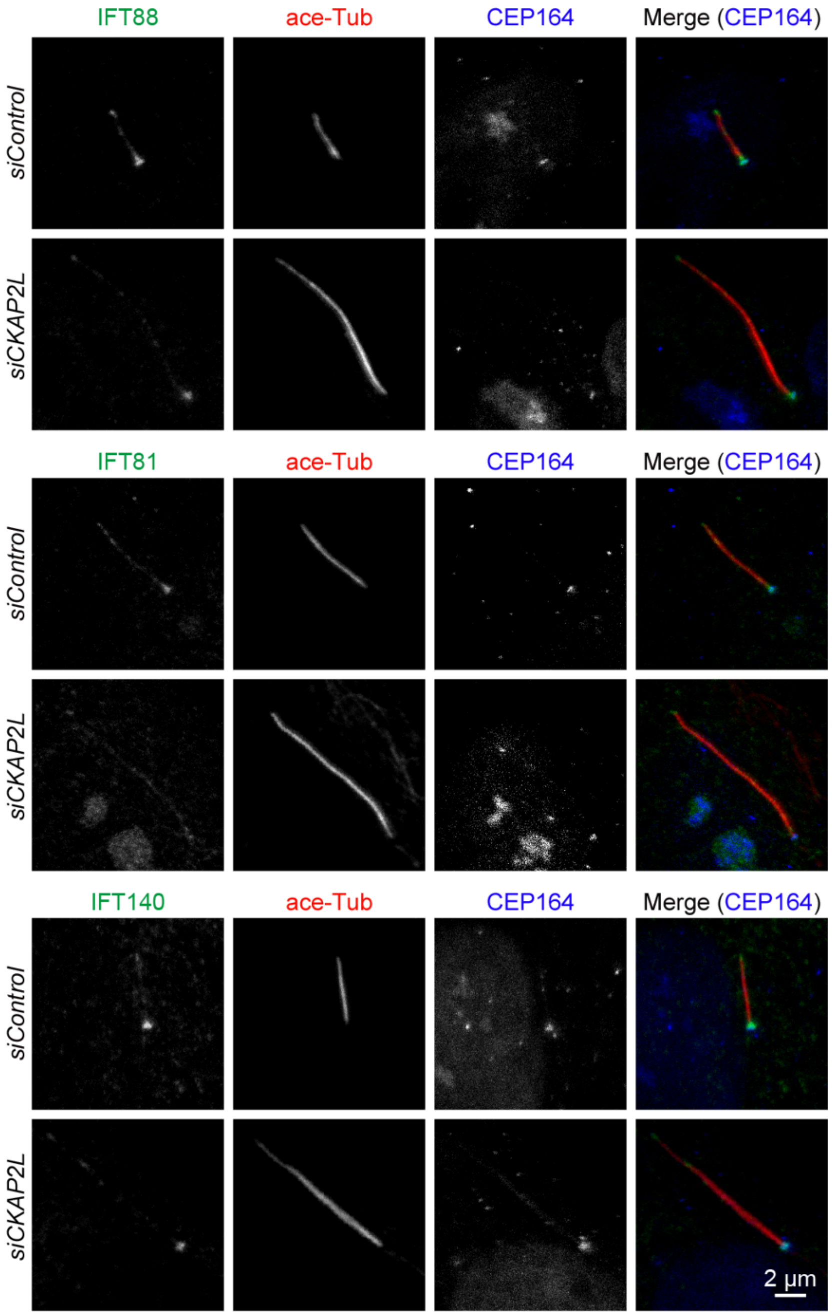

Supplementary Figure S2 CKAP2L does not alter the ciliary localization of IFT machinery. Immunofluorescence images of RPE1 cells treated with siControl or siCKAP2L oligo. Cells were stained for the IFT-B components IFT88 and IFT81, and the IFT-A component IFT140 (green). The ciliary axoneme is marked with acetylated-tubulin (ace-Tub; red) and the basal body with CEP164 (blue).

Supplementary Tables

Supplementary Table S1 List of the plasmid and primers used.

**Plasmid**	**Insert**	**Amino acids**	**GenBank**
pDONR-CKAP2L-rescue	CKAP2L	1–745 aa	NM_152515
	**Primers**	**Sequence (5'→3')**	
Genotyping	*Ckap2l*KO-F1	GCCATCTCACCAGCCCAATT	
	*Ckap2l*KO-R1	TTCCTGACTGCTCTGCCTTTCC	
	*Ckap2l*KO-F2	CCCCATGTTGCAATGTTCTCTC	
	*Ckap2l*KO-R2	CTTGACGTGAGTCCTCTGCTT	
qPCR	*Ckap2l*-F	CACAGAAGTAACCTCTGACACC	
	Ckap2l-R	GGCATCCCGCATATTCTAGG	
siRNA-CKAP2L	*Sense*	CAAAGuuGuuGGCAAGTAAtt	
	Antisense	uuACuuGCCAACAACuuuGtt	
siRNA-Ckap2l#1	*Sense*	GACCUCCCAUGAAAUUUAATT	
	Antisense	UUAAAUUUCAUGGGAGGUCTT	
siRNA-Ckap2l#2	*Sense*	GGGUGUACUUCCUAAUGAATT	
	Antisense	UUCAUUAGGAAGUACACCCTT	

Supplementary Table S2 List of antibodies used.

Primary antibodies					
**Antigen**	**Isotype**	**Supplier**	**Cat. #**	**WB**	**IF**
Rat anti-GFP antibody	IgG2a,k	BioLegend	338002	1:10000	1:200
Rabbit anti-CEP164 antibody	IgG	Proteintech	22227-1-AP		1:500
Rabbit anti-ARL13B antibody	IgG	Proteintech	17711-1-AP		1:1000
Rabbit anti-CKAP2L antibody	IgG	Proteintech	17143-1-AP	1:1000	
Mouse anti-Alpha tubulin antibody	IgG1	Abcam	ab7291		1:1000
Rabbit anti-Pericentrin antibody	IgG	Abcam	ab4448		1:1000
Rabbit anti-CKAP2L antibody	IgG	Abcam	ab221897		1:200
Mouse anti-γ-Tubulin antibody	IgG1	Sigma	T6557	1:2000	1:500
Rabbit anti-Cyclin B1 antibody	IgG	ABclonal	A22435	1:1000	
Mouse anti-Acetyl-Tubulin antibody	IgG1	Proteintech	66200-1-Ig		1:500
Rabbit anti-NEDD1 antibody	IgG	Proteintech	13993-1-AP	1:1000	
Rabbit anti-IFT88 antibody	IgG	Proteintech	13967-1-AP		1:200
Rabbit anti-IFT81 antibody	IgG	Proteintech	11744-1-AP		1:200
Rabbit anti-IFT140 antibody	IgG	Proteintech	17460-1-AP		1:200
Guinea pig anti-CEP164 antibody	IgG	home-made			1:1000
**Secondary antibodies**					
**Name**	**Label or dye**	**Supplier**	**Cat. #**	**WB**	**IF**
Goat anti-Mouse IgG (H+L)	HRP	Invitrogen	31430	1:20000	
Goat anti-Rabbit IgG (H+L)	HRP	Invitrogen	31460	1:20000	
Donkey anti-Guinea pig IgG (H+L)	Alexa Fluor 647	Jackson ImmunoResearch	706-605-148		1:1000
Donkey anti-Guinea mouse IgG (H+L)	Alexa Fluor 555	Jackson ImmunoResearch	715-565-151		1:1000
Donkey anti-Rabbit IgG (H+L)	Alexa Fluor 647	Thermo Fisher	A32795		1:1000
Donkey anti-Rat IgG (H+L)	Alexa Fluor 488	Thermo Fisher	A48269		1:1000
Donkey anti-Rabbit IgG (H+L)	Alexa Fluor 488	Thermo Fisher	A32790		1:1000
Donkey anti-Mouse IgG (H+L)	Alexa Fluor 488	Thermo Fisher	A32766		1:1000
Donkey anti-Rabbit IgG (H+L)	Alexa Fluor 555	Thermo Fisher	A32794		1:1000

Supplementary Movie

Supplementary Video S1 Sperm motility in representative regions of WT and *Ckap2l* KO spermatozoa.

Mature spermatozoa released from the cauda epididymides of 8-week-old WT and *Ckap2l* KO mice were live-imaged with the computer-assisted sperm analysis (CASA) image system (IVOS II, Hamilton Thorne). Image sequences are played back at 5 fps.
